# Catheter Ablation of Ventricular Tachycardia Originating from the Left Posterior Papillary Muscle Guided by the Shadow of a Multipolar Catheter

**DOI:** 10.1016/s0972-6292(16)30525-3

**Published:** 2012-07-28

**Authors:** Shiro Nakahara, Noritaka Toratani, Kan Takayanagi

**Affiliations:** Department of Cardiology, Dokkyo University Koshigaya Hospital, Saitama, Japan

**Keywords:** ventricular tachycardia, papillary muscle, radiofrequency catheter ablation

## Abstract

A 62-year-old man without structural heart disease underwent electrophysiological testing for ventricular tachycardia (VT). Hemodynamically unstable VT was induced after isoproterenol (ISP) provocation. Electroanatomical mapping using a multipolar catheter identified the earliest activation originating from the posterior papillary muscle (PPM) where prepotentials preceding the local ventricular electrogram were observed. Irrigated radiofrequency current guided by the shadow of a multipolar catheter eliminated the VT. This case suggested that multipolar catheters may be helpful for identifying tachycardia origins arising from the PPM.

## Introduction

It has been reported that catheter ablation of idiopathic focal ventricular arrhythmias originating from papillary muscles (PAMs) is challenging . The complex structure of the PAMs may cause difficulty with the catheter manipulation during mapping and ablation. We report a case of a ventricular tachycardia (VT) originating from the LV posterior PAM (PPM), which was successfully eliminated by catheter ablation guided by the shadow of a multipolar catheter.

## Case

**Figure 1:**
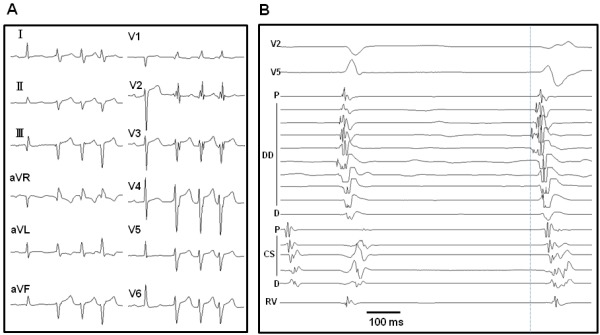
Preprocedural 12-lead ECG recorded during the VT (A), and intracardiac tracings recorded during the PVCs (B). The 12-lead ECG revealed incessant bursts of VT with a right bundle branch morphology and left axis deviation. The prepotential recorded at the mid-portion of the duodecapolar catheter preceded the QRS onset by -22ms. DD = duodecapolar catheter; CS = coronary sinus catheter.

**Figure 2:**
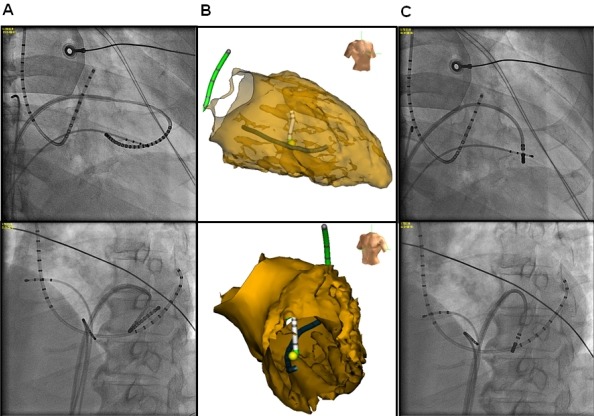
Right (upper panel) and left (lower panel) anterior oblique fluoroscopic views (A and C) and electroanatomical map images (B). The duodecapolar catheter is positioned around the posterior papillary muscle (A), and the ablation catheter is positioned at the earliest activation site of the PVCs around the distal portion of the posterior papillary muscle (C) guided by the shadow of the duodecapolar catheter (B).

A 62-year-old man was admitted to our hospital for catheter ablation of premature ventricular complexes (PVC). The 12-lead electrocardiogram (ECG) recorded during the PVCs exhibited a QRS with a right bundle branch block morphology and left axis deviation, consistent with a posterolateral left ventricular (LV) origin. A cardiac computed tomography (CT) scan was obtained 1 day before the procedure. After left ventriculography, a transseptal puncture was performed for LV mapping. The LV geometry was created using a duodecapolar catheter (Livewire, 2-2-2mm spacing, St. Jude Medical, Minnetonka, MN) guided by an LV angiogram for the integration of CT images into the NavX system using Fusion (Ensite, St. Jude Medical, Minnetonka, MN). This system allowed the visualization of a multipolar catheter, and the outline of the complex structure of the papillary muscle (PAM) integrated into the CT images. Programmed ventricular stimulation did not induce any PVCs. After provocation with isoproterenol (ISP), frequent monomorphic PVCs and nonsustained VT with a QRS morphology similar to that of the PVCs emerged (Figure 1A). However, the blood pressure decreased due to the effect of the ISP and the emergence of nonsustained VT. LV activation mapping using the duodecapolar catheter was performed during the hemodynamically unstable condition. The duodecapolar catheter was suitable for recording the local electrograms preceding the QRS onset of the PVCs around the posterior PAM (PPM) region, and identified the site of the earliest ventricular activation at the mid portion of the PPM (Figure 2A,B) which preceded the onset of the QRS complex by -22 ms (Figure 1B). The earliest site of the targeted PVC was tagged on the integrated CT images with a shadow of the duodecapolar catheter because it seemed difficult to maintain stable contact with the ablation catheter tip at the earliest site during the hemodynamically unstable condition. After discontinuing the ISP infusion, the duodecapolar catheter was removed from the LV, and the ablation catheter was moved to the earliest activation site of the PVCs guided by the shadow of the duodecapolar catheter despite the disappearance of the PVCs (Figure 2C). Good pacemapping was obtained at the earliest activation site of the PVCs, and irrigated RF energy was delivered at that site. During the catheter ablation, irrigated radiofrequency (RF) current was delivered in the power control mode starting at 30W with an irrigation flow rate of 30 mL/min. The RF power was titrated to as high as 50W, with the goal of being able to achieve a decrease in the impedance of 10 Ω and with care taken to limit the temperature to < 41º. Ventricular responses were documented during the RF application. No ventricular arrhythmias emerged after the ISP infusion, and by ten months after the procedure, the patient had no further recurrences of the tachyarrhythmia.

## Discussion

Recently, the PAMs in the LV have been reported to be arrhythmogenic in human hearts after a myocardial infarction. In addition, idiopathic focal ventricular arrhythmias have been reported to originate from both the posterior and anterior PAM in the LV. In identifying the origin of idiopathic LV ventricular arrhythmias (VAs) arising from a PAM, activation mapping is the most reliable method, although pace mapping usually provides helpful clues. The use of noncontact mapping for idiopathic LV tachycardias was also reported, however no reports so far are available regarding the use of mapping for PAM VAs. Noncontact mapping may have been suitable in this case, because the conventional point by point mapping was difficult due to the hemodynamically unstable condition. In this case, contact mapping using a multipolar catheter identified the site of the earliest ventricular activation at the mid portion of the PPM. A low amplitude prepotential which preceded the QRS onset at the successful ablation site on the PPM was recorded whereas a previous study reported that a Purkinje potential often preceded the QRS onset at the successful ablation site of PAM VAs. There is still controversy as to whether a Purkinje potential always can be recorded at the successful ablation sites of PAM VAs. This discrepancy may be explained by the heterogeneity of the VAs ablated around the PAM, with some being endocardial (close to the Purkinje fibers) and other intramyocaridal. It also should be noted that the use of an irrigated catheter, as was used in this case, has been required to achieve a lasting ablation of PAM origin VAs because the origin is deep relative to the endocardial surface of the PAMs.

RF catheter ablation of idiopathic LV PAM VAs is particularly challenging. To the best of our knowledge, this is the first report describing the efficacy of a multipolar catheter for mapping around the PPM region. The anatomically complex structure of the papillary muscle complicates the manipulation and stability of catheters during unstable hemodynamic conditions during an ISP infusion. This case suggested that the multipolar catheter may be helpful for predicting the earliest site of VAs arising from the PPM.

## References

[R1] Doppalapudi H (2008). Ventricular tachycardia originating from the posterior papillary muscle in the left ventricle: A distinct clinical syndrome. Circ Arrhythm Electrophysiol.

[R2] Yamada T (2009). Idiopathic focal ventricular arrhythmias originating from the anterior papillary muscle in the left ventricle. J Cardiovasc Electrophysiol.

[R3] Bogun F (2008). Post-infarction ventricular arrhythmias originating in papillary muscles. J Am Coll Cardiol.

[R4] Yamada T (2009). Real-time integration of intracardiac echocardiography and electroanatomic mapping in pvcs arising from the lv anterior papillary muscle. Pacing Clin Electrophysiol.

[R5] Yamada T (2010). Electrocardiographic and electrophysiological characteristics in idiopathic ventricular arrhythmias originating from the papillary muscles in the left ventricle: Relevance for catheter ablation. Circ Arrhythm Electrophysiol.

[R6] Chen M (2005). Non-contact mapping and linear ablation of the left posterior fascicle during sinus rhythm in the treatment of idiopathic left ventricular tachycardia. Europace.

[R7] Good E (2008). Ventricular arrhythmias originating from a papillary muscle in patients without prior infarction: A comparison with fascicular arrhythmias. Heart Rhythm.

